# Patient and Family Preferences on Health System-Led Direct Contact for Cascade Screening

**DOI:** 10.3390/jpm11060538

**Published:** 2021-06-10

**Authors:** Nora B. Henrikson, Paula Blasi, Marlaine Figueroa Gray, Brooks T. Tiffany, Aaron Scrol, James D. Ralston, Stephanie M. Fullerton, Catherine Y. Lim, John Ewing, Kathleen A. Leppig

**Affiliations:** 1 Kaiser Permanente Washington Health Research Institute, Seattle, WA 98101, USA; paula.r.blasi@kp.org (P.B.); marlaine.s.figueroagray@kp.org (M.F.G.); brooks.t.tiffany@kp.org (B.T.T.); aaron.scrol@kp.org (A.S.); james.d.ralston@kp.org (J.D.R.); research@catherinelim.com (C.Y.L.); john.j.ewing@kp.org (J.E.); 2 Department of Bioethics and Humanities, University of Washington School of Medicine, Seattle, WA 98195, USA; smfllrtn@uw.edu; 3 Kaiser Permanente Washington, Seattle, WA 98101, USA; kathleen.a.leppig@kp.org

**Keywords:** cascade screening, genetic testing, family communication, precision medicine, ELSI

## Abstract

Health benefits to relatives of people at known genetic risk for hereditary cancer syndromes is key to realizing the promise of precision medicine. We conducted a qualitative study to design a patient- and family-centered program for direct contact of relatives to recommend cascade genetic testing. We conducted two rounds of data collection using focus groups followed by individual interviews with patients with HBOC or Lynch syndrome and a separate sample of people with a family history of hereditary cancers. Results indicate that U.S.-based health system-led direct contact of relatives is acceptable to patients and families, should take a programmatic approach, include consent of relatives before proband testing, complement to existing patient-mediated disclosure, and allow for relative control of information. Our findings suggest a set of requirements for U.S.-based direct contact programs that could ultimately benefit more relatives than current approaches.

## 1. Introduction

Health benefits to relatives of people at known genetic risk for hereditary cancer syndromes is key to the promise of precision medicine. Key to this is cascade screening—case finding in people with known pathogenic variants. Currently in the United States, the responsibility for notifying family members of potential risk falls to the individual patients [1]. Each family member then assumes responsibility for initiating their own genetic testing or follow-up care. However, up to a third of at-risk relatives may go un-notified [2,3,4,5], representing a missed opportunity to benefit from genetic counseling, testing, and appropriate clinical follow-up [6]. In our own institution, two of three patients with actionable genetic test results reported only partial disclosure to family members [7].

Data from other countries with industrial economies suggest that direct contact programs—where health systems or providers conduct risk disclosure directly to family members—may be the most effective method of relative notification, but such programs have not been implemented in the U.S. Furthermore, our pilot work revealed a lack of clarity in how to develop and implement a family-based program in a system designed for individual patient care [8]. We conducted a qualitative human-centered design study to draft a set of requirements for direct contact of relatives to recommend cascade testing that was consistent with patients’ and families’ needs and preferences.

## 2. Results

The total sample size included 12 probands and 46 people with a family history of cancer (*n* = 58). All participants were members of Kaiser Permanente (KP) Washington. The sample was predominantly female (64%), employed (69%), and married (69%). There was more racial/ethnic and educational diversity in the relative sample than in the proband sample. Consistent with our inclusion criteria, all participants reported either first- or second- degree relatives with a history of hereditary breast and ovarian cancer syndrome or Lynch syndrome or family history of breast, ovarian, uterine, pancreas, colorectal or other genitourinary cancers; 28% also reported a personal history of cancer (Table 1).

We were limited by the size and diversity of the population for probands, but our relative sample was more balanced in terms of sex, race/ethnicity, and education. The proband sample was predominantly female (75%), white (92%), and over age 50. The relative sample was 62% female, 50% people of color, and mean age 50 (range 19–82). Compared to the proband sample, relatives were more likely to be working (74% vs. 50%), and less likely to have a personal history of cancer (17% vs. 67%).

Participant-reported experiences with genetic testing included genetic testing as part of their own cancer diagnosis, following a relative’s cancer diagnosis, or no experience with genetic testing. Among participants who shared their personal or family history with genetic testing, experiences ranged from feeling positive and well-supported, to feeling uncertain about the significance of their results, to feeling anxious and worried. Experiences with genetic counseling were generally positive.

Participants also discussed their personal experiences with sharing genetic test results among family members. Some participants said their families were very open about sharing genetic test results and other health information. Others cited challenges sharing such information in their families due to family tensions (“dysfunctional”) or being out of contact with relatives; relatives not wanting to discuss genetic risk; patients wanting to protect their own or relatives’ privacy; concerns about worrying a relative, and uncertainties about how to address relatives’ potential privacy concerns and fears about insurance discrimination.

### 2.1. Preferences about Direct Contact Programs

Table 2 summarizes the four themes we identified about general patient and family preferences with respect to direct contact programs, with exemplar quotes. First, the most commonly cited benefit of direct contact was relatives’ awareness of increased disease risk, particularly if that information could help detect disease at an earlier stage or prevent cancer. Participants also mentioned how genetic test results might prompt someone to make health behavior changes that could reduce cancer risk or inform reproductive decision-making. Also mentioned was the possibility that health system-led direct contact could provide the most accurate information to relatives as quickly as possible. Participants drew clear distinctions between actionable risk information (such as enabling increased surveillance or testing, or encouraging healthy lifestyle behaviors) versus non-actionable information (e.g., false paternity, diseases like Huntington where there is no intervention).

Our second theme reflects participants’ nuanced discussions of the health system’s role in risk notification, which was a new idea for most participants. Participants were supportive of direct contact programs based on the potential health benefits to relatives. However, participants engaged in rich focus group discussions about whether notifying relatives should remain the patient’s responsibility and decision; weighing the potential benefits of direct contact programs against potential privacy infringements; and respecting relatives’ rights not to be notified or to find out risk information. These conversations were consistent across groups and education level of participants.

The third theme was about how direct contact should be handled programmatically. Participants felt that direct contact of relatives should be a program or initiative of the organization, and not an individual clinician’s responsibility. Participants were protective of the role and time of the clinician and suggested looking to other disease management programs for guidance. Of note, participants in every focus group organically derived the idea of pre-consenting relatives, in which either the relative or the patient would provide broad consent to participate in a direct contact program well before a proband’s testing occurred. Participants became actively engaged in pre-consent as a solution to the potential privacy infringements, particularly relatives’ right not to know. They suggested specific ways in which pre-consent could be implemented, including at the time of enrollment in KP, before genetic testing occurs, or during a routine medical appointment. They also suggested these preferences could be regularly updated in the same way that patient contact information is regularly updated.

Our fourth theme regarded placement of direct contact in the context of the current standard of care. Despite support for direct contact programs, many participants considered them a complement to, not a replacement of, the current standard of patient-led contact. Some people felt that familial communication was primarily the patient’s obligation, and that direct contact should only occur if the patient was unable to contact their own relatives. This was particularly endorsed in the proband focus group, many of whom reported positive experiences with genetic counseling and sharing results with their own relatives. However, a range of preferences was expressed on this point, as others felt that direct contact should be a more routine part of care.

### 2.2. Requirements for Direct Contact Programs

We also identified a series of design requirements implied by the themes above. Informed by patient and family preferences, these requirements can guide implementation of health system-led direct contact programs. The requirements maximize the rights of the relative, outline clear consent needs, and suggest specific communication points and methods. The requirements and exemplar participant quotes are listed in Table 3.

**Patients should provide consent for a provider to contact their relatives**. Participants were very clear that the patient should provide permission for their relatives to be contacted. People appreciated that logistically, the participant would typically be the source of the relatives’ contact information, so discussion on this point was limited.

**Relatives should be able to decide which (if any) information they want to receive, and how they act on that information**. Recognizing that direct contact programs would often entail a relative getting a “cold call” from a possibly unfamiliar health system or clinician, participants consistently advocated for relatives’ right to have multiple points to opt out of the disclosure process. This would likely take the form of a multi-step contact process, such as an initial contact, followed by a conversation, and only then followed by the results disclosure itself. Participants felt strongly opposed to the idea of a single direct disclosure (e.g., a mailed letter with the patient’s genetic test results), citing that this might violate a relatives’ right not to know such information. There was limited discussion on how clinician-to-clinician communication should or would occur, but participants felt that relatives should also provide consent to any placement of risk information in their medical record.

There was consistent support for obtaining relatives’ consent to be contacted before testing occurs. Participants suggested specific times when this pre-consent of relatives could be implemented, including at the time of enrollment in KP, immediately before genetic testing of the proband, or during a routine medical appointment. Participants also suggested processes for updating preferences, such as when the health system routinely asks for updates to one’s contact information.

Multiple communication channels and multiple efforts should be used to communicate with relatives, with clear communication of the final outreach. Preferred communication channels varied, and participants expressed neither strong nor consistent opinions about whether phone, email, secure message, or letter would be the best way to make the initial direct contact. Some people mentioned that the age of the relative would determine the best method of outreach (e.g., using email to reach younger relatives). Participants supported trying to reach individual relatives in multiple ways, yet thought the final outreach attempt should be clearly noted.

**Communication points with relatives**. Specific communication points included clear communication of the proband’s consent to contact them, whether or not the proband is named; the reason for the direct contact, and information on the potential inherited risk and associated diseases. Cost and coverage information and information about privacy and genetic discrimination laws were also mentioned, but less frequently.

**The clinician should make a clear recommendation for genetic testing and provide clear follow-up steps**. Though preferences varied for modes of initial contact, participants expressed a strong preference that a verbal conversation with a clinician was important for the actual risk notification discussion. The clinician conducting the verbal conversation should make a clear recommendation for or against genetic testing and provide clear steps for how to obtain testing.

**Patients should still receive support or written resources to share with their relatives.** Consistent with the theme that direct contact adds to, but does not replace, patient-led contact, participants suggested tools and resources that the care team could provide to support patient sharing of genetic test results with relatives. These included a letter from the doctor and a packet of written materials about the test results and the genetic condition, with referral information for a doctor or genetic counselor and resources on health insurance coverage options for genetic testing. Probands who had already been through genetic counseling and testing reflected positively on the resources they had received during their genetic counseling as aids to help with patient-led disclosure.

## 3. Discussion

We conducted a qualitative human-centered design study to co-design a patient- and family-centered program for direct contact of relatives to recommend cascade genetic testing for HBOC or Lynch syndrome. Our work suggests that direct contact programs are an acceptable complement to existing patient-mediated U.S.-based cascade screening efforts. However, privacy protections and relative control over information flow are likely keys to optimizing acceptability. A programmatic approach that included broad consent of relatives before proband testing held particular interest for participants as a way to mitigate concerns about privacy, consent, and right not to know.

Direct contact-based cascade screening programs are novel in U.S. settings. A growing body of evidence suggests that health system-led direct contact of relatives is acceptable to clinicians and patients and is likely to be more successful than patient-led contact alone. Two systematic reviews have found that direct contact approaches were associated with greater cascade screening uptake. One also found that suboptimal communication between patient and relatives was a major barrier to cascade screening effectiveness [9]. The other review examined 15 studies and found that uptake of pre-symptomatic genetic testing with patient-mediated approaches was considerably lower than with health systems-led approaches [10]. For example, a study conducted in an Australian familial cancer service [11] compared patient-led notification to direct outreach by the cancer service. At 2 years follow-up, 40% of relatives from the direct outreach group had received genetic testing and had their genetic status defined compared to 23% of relatives from the comparison group (*p* < 0.001). In the intervention group, 25 people (7% of relatives in the intervention arm) declined to participate; no complaints were received about privacy concerns by the study team or to their institution [11]. Other studies exploring direct outreach to relatives have found similar results and acceptability levels among patients in Finland [12] and the United Kingdom [13].

Evidence primarily from outside the U.S. suggests that direct contact programs are also acceptable to patients. A review of 32 qualitative and survey studies found that that direct contact programs were largely acceptable and not associated with harms, though they were less studied than patient-mediated approaches [14]. A 2020 cross-sectional population-based survey conducted in Sweden found that people overwhelmingly would want to be notified of a moderate (89.2%) or high (90.6%) hereditary risk of colorectal cancer; the majority of respondents preferred to be informed by a health care professional (80.1% and 75.5%) rather than by a family member (18.1% and 20.1%) [15]. An evaluation of familial hypercholesterolemia cascade screening in the Netherlands found that being directly approached was broadly acceptable to relatives and resulted in high acceptance of screening [16]. In cross-sectional and qualitative studies, patients have conceptualized genetic information as familial, not simply individual, and report willingness to have providers notify relatives directly [17,18,19,20,21].

Resistance to direct contact programs in U.S. settings is likely attributable at least in part to the sacrosanct nature of the dyadic clinician-patient relationship, a widely accepted ethical foundation in U.S. medical practice. However, despite legal protections that relieve them of obligations to contact relatives directly, healthcare providers often report a personal sense of duty to their patients’ relatives. A survey of U.S. genetic counselors found that 46% of counselors had encountered cases where a patient refused to inform a relative of their risk. A higher proportion (63%) reported agreement with the idea that genetic counselors have an obligation to inform at-risk relatives [22]. A follow-up study found that 69% of medical geneticists reported an obligation to notify their patients’ relatives of a potential genetic risk [23].

Ethical arguments increasingly argue for a general clinician responsibility towards at-risk relatives and toward more family-level obligations, particularly in the context of shared genetic information [24,25,26,27]. The HIPAA privacy rule permits several avenues for direct clinician contact of at-risk relatives, including and most easily with patient consent [28].

However, despite reporting an obligation to at-risk relatives, clinicians may have concerns about implementation, noting the ethical tensions between individual and family interests and interpretation of duty considering the heterogeneity of conditions served by genetic services [29,30,31]. This tension suggests a further rationale for an active role by health systems in supporting outreach to family members, and is supported by our data, where participants thought that direct contact should be a programmatic effort rather than an individual clinician task.

Our findings suggest that patient and family preferences support the existing literature suggesting that direct contact programs are acceptable, in line with HIPAA guidance, and consistent with a programmatic approach.

As a result of this research, we designed a direct contact program consistent with this guidance and we are currently testing it in a feasibility trial. Furthermore, we found human-centered design to be a valuable method for rapidly and meaningfully eliciting needs and preferences for a novel program in U.S. settings. In particular, it helped us anticipate the relative’s experience of being directly contacted and to design requirements for such programs that are aligned with relatives’ preferences. Participants demonstrated nuanced understanding of the tradeoffs between privacy and potential health benefits for these types of programs. Participants’ suggestion that relatives be able to provide very early broad consent provides an important early anchor in how to disclose results to relatives.

Limitations of this study include the use of a single health system for data collection, and the setting within an integrated health system. However, we intentionally recruited a sample of economic, educational, and racial/ethnic diversity to gain a deep understanding from a broad sample. Furthermore, integrated health systems are an ideal setting in which to implement direct contact programs, since they provide care for entire families, often multiple generations, and may have access to infrastructure to implement such a program. In addition, we used rapid analysis techniques, which may have introduced some bias in interpretation compared to more traditional qualitative analyses, but are consistent with those typically used within human-centered design. Furthermore, we conducted two rounds of data collection, which allowed for confirmation of findings over time.

Future research should include replication of our findings over other geographic areas, care delivery settings, and populations. Research will also be needed to fully explore the reimbursement and policy changes that might be needed from a payer perspective to implement direct contact programs in the United States.

In conclusion, our study provides novel, pragmatic information on how to design a patient- and family-centered direct contact cascade screening program for U.S. settings. In particular, attention to relative preferences for information flow, consent to be contacted before proband testing, clear communication with relatives focusing on the benefits of cascade screening, and continued support of patient-mediated disclosure may be key to successful direct contact programs. Our findings suggest design requirements for U.S.-based direct contact programs that could help to reach, and ultimately benefit, more relatives than current approaches, moving toward delivering on the promises of precision medicine.

## 4. Materials and Methods

The study setting was Kaiser Permanente Washington, an integrated health system in Washington State that provides care for more than 700,000 members.

### 4.1. Eligibility Criteria and Sampling

The study population was either people who had received genetic counseling and testing at KPWA Clinical Genetics (“probands”) or KPWA members reporting a family history of cancer (“relatives”). We identified probands using records kept by KPWA Clinical Genetics. Inclusion criteria included age 18–70 at time of test result; currently enrolled and still living; tested at KPWA since 2004 and found to be carrying known pathogenic variants or variants of uncertain significance (VUS) in the *BRCA1*, *BRCA2*, *MLH1*, *MSH2*, *MSH6*, or *PMS2* genes. People receiving care in KPWA’s external contracted network were excluded.

To get perspectives from different families, relatives were not necessarily related to probands, but a separate sample of people with family history of cancer. For relatives, we sampled an intentionally broad random sample using administrative data. Inclusion criteria were age 18–70 at sampling, currently enrolled and receiving care at one of three KPWA owned-and-operated clinics in Western Washington (chosen to maximize geographic and rural/urban diversity), and self-reported family history of breast, ovarian, uterine, pancreas, colorectal or other GU cancers in at least one first- or second-degree relative that was entered in the medical record.

For probands, we recruited from the sampling frame until 30% of the sample was male. Additionally, we sought to include both HBOC and Lynch syndrome patients, at least 1 person with a VUS result. For relatives, we used a consecutive sampling approach to recruit an equal mix of both males and females and people both under and over age 50. We purposively sampled to ensure at least half were from non-White non-Hispanic backgrounds and a distribution of people who had not attended college, to increase the socioeconomic and racial/ethnic diversity of the sample. Since we were designing a process for informing relatives, we intentionally oversampled due to prior studies showing racial and ethnic differences in knowledge of genetic testing and concerns about potential misuse of genetic testing [32].

We were limited in racial/ethnic and educational distribution by the smaller size of the proband sample, but for the relative sample, we intentionally oversampled until a racially and educationally diverse sample was achieved. Additionally, for the proband sampling, we sought to include both HBOC and Lynch syndrome patients, and at least 1 person with a VUS result.

We mailed a letter to potentially eligible participants that described the study and provided both a phone number to call to opt in or opt out and a statement that a study team member may call them. To reach the targeted sample characteristics, we called potentially eligible participants, completed eligibility assessment, and enrolled participants consecutively until our targeted distribution was reached.

### 4.2. Data Collection

We conducted two rounds of qualitative design sessions. The first round of sessions (Round 1) used focus group design methods, and the second used individual, video interview design methods. We modeled these focus groups after the Future Workshop, a participatory design format focused on imagining different future states that represent a substantial departure from the current state. The format consists of three main steps: critiquing the present, envisioning the future, and implementing potential ideas. This method can be useful when asking people to imagine future states that represent a substantial departure from the current state [33,34]. In the second round, we sought participant feedback on a draft direct contact process we developed based on Round 1 findings.

**Figure 1:**
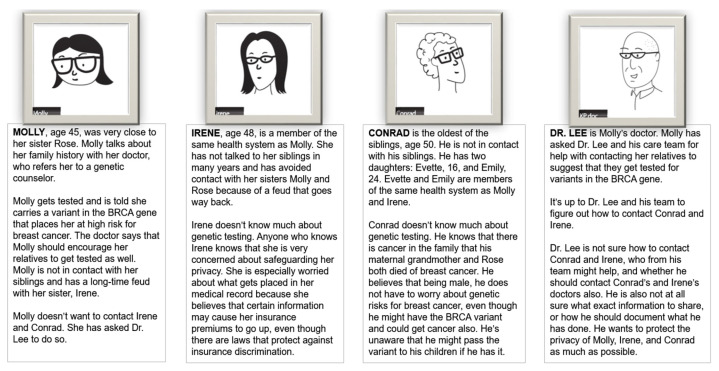
Fictitious family and scenario used for discussion with participants during focus groups and interviews.

For both design rounds, we created and presented to participants a fictitious family whose members represent known issues with communicating genetic risk information—difficulty communicating with estranged relatives and communicating complex genetic information to one’s relatives (Figure 1). This was to encourage participants to collectively critique the same problems. We created cards with descriptions of each family member’s story. We asked participants to volunteer to read those descriptions and introduce each family member at the start of the workshop. The cards are a way to create “agonistic public spaces” for investigating social and ethical dilemmas from multiple perspectives and to encourage participants empathize with the perspectives and preferences of various characters in co-designing a potential solution [35,36].

#### 4.2.1. Round 1 Design Groups: Future’s Workshops (*n* = 29 Relatives, *n* = 8 Probands)

The goal of the first round was to co-develop a direct contact process with participants. We started each focus group by presenting a fictitious family based on real clinical cases in which patients had not informed their relatives about genetic test results. We then explained the current state—patient-mediated risk notification—and led a discussion of the implications of incomplete disclosure by relatives. We presented participants with a partial storyboard (similar to a comic strip) to complete, depicting a patient who asks a physician to contact her relatives directly. Participants worked in pairs to envision the rest of the story and sketch out a potential future process of direct contact, and then shared and discussed their storyboards with the full group.

Each focus group was attended by two study team members; one served as facilitator and one as notetaker. All focus groups discussions were audio-recorded and professionally transcribed, and all participant-created storyboards were scanned and stored as PDF files. For analysis of Round 1 data, we used template analysis, a deductive form of rapid qualitative analysis [37,38]. We created a draft codebook based on our research questions and interview guide. After piloting the codebook and revising accordingly, we iteratively coded both the transcripts and the participant-completed storyboards, adding inductive codes as they emerged from the data. Through a series of team analysis sessions, we drafted a familial notification process that we would present to participants in round 2.

#### 4.2.2. Round 2 Design Interviews: Clarification of Patient and Relative Needs (*n* = 17 Relatives, *n* = 4 Probands)

**Figure 2:**
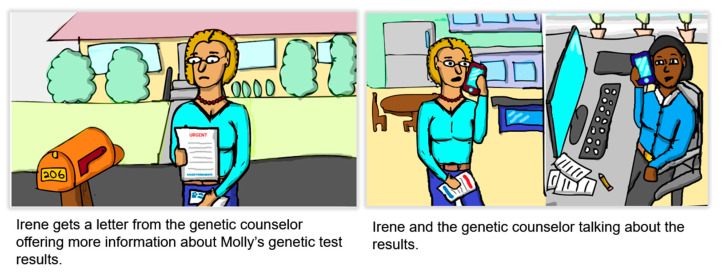
Screen shots of narrated video used in Round 2 depicting the draft direct contact program.

Round 2 included different participants than Round 1. Based on analyses of Round 1 future workshops, we developed a video version of the draft direct contact process and shared it with participants during the call (Figure 2 shows an example). We then asked a series of questions to debrief each section of the video. We also included additional specific questions designed to elicit information that was not clear in the first round (e.g., whether and how to use the patient’s name when contacting relatives).

Analysis for Round 2 was designed to be confirmatory and to identify new information or preferences not identified during Round 1. As in Round 1, we used template analysis. We created an episode profile to summarize each interview, with a focus on identifying new information or preferences that were not identified in Round 1. After analyzing these data in a series of team analysis meetings, we revised and finalized our draft requirements for health system-led direct contact programs.

## Figures and Tables

**Table 1:** Sample demographics.

Combined Round 1 (*n* = 29 Relatives, *n* = 8 Probands) and Round 2 (*n* = 17 Relatives, *n* = 4 Probands)	Relatives	%	Proband	%
*n*	46		12	
Female	28	61%	9	75%
Age, mean years (range)	50.1 (19–82)		61.2 (34–77)	
Age group				
18–29	6	13%	0	0%
30–39	8	17%	2	17%
40–49	7	15%	0	0%
50–59	12	26%	3	25%
60–69	10	22%	3	25%
70+	3	7%	4	33%
Race/ethnicity (not mutually exclusive)				
Hispanic	2	4%	0	0%
White	23	50%	11	92%
Black or African-American	9	20%	1	8%
Asian	4	9%	0	0%
American Indian/Alaska Native	5	11%	0	0%
Other	4	9%	0	0%
Education				
Some high school	2	4%	0	0%
High school or GED	8	17%	0	0%
Some college	17	37%	0	0%
4-year college degree	9	20%	2	17%
Post-graduate	10	22%	10	83%
Employment				
Working	34	74%	6	50%
Retired	10	22%	6	50%
Marital status				
Married	31	67%	9	75%
Divorced	1	2%	0	0%
Single	10	22%	2	17%
Widowed	4	9%	1	8%
Cancer history				
Personal history of cancer	8	17%	8	67%
Family history of cancer				
First degree relative	37	80%	12	100%
Second degree relative	44	96%	12	100%

**Table 2:** Themes: patient and family preferences for direct contact programs.

Theme	Exemplar Quote
The potential health benefits to relatives is the main rationale for direct contact programs.	There might be a small percentage who may be feeling like I verbalized, that they don’t want to—they don’t even want to carry the burden of knowing that there is a possibility of something. But for a huge portion of the population, I would think, they would just want to know. And then they can go talk to their private provider and then go on from there. I would want to know. I would want to know. Regardless of the feelings I may have, ultimately after I calm down, I would want to know if I had a greater risk of dying like my sister did at 41. (relative)
Participants were supportive of direct contact, but aspects of direct contact programs were new and raised concerns about whose duty it is to notify relatives and about how privacy would be maintained.	SPEAKER 1: Something that came to my mind was the ethical situation. Where the doctor knows that someone is at high risk, I mean, shouldn’t they contact somebody? …It’s not protecting Molly’s privacy. It’s protecting Irene’s or Conrad’s or Tina’s or whosever privacy. 1 …It’s a tradeoff, the privacy versus the health—the lifesaving information. That’s what it amounts to, doesn’t it? (SPEAKER 2): But I—maybe. But I think we’re forgetting that, like, Molly can also reach out. It’s not, like, if the doctor doesn’t do it, they’re not going to get this information. (proband)
Participants thought direct contact should be a program, not an individual provider’s responsibility. Pre-consenting programs were frequently suggested.	This doctor doesn’t have time. He does his job. Every patient is important to him, but it’s not his job to call or write letters or send emails.1 (relative) I kind of wonder if, like, if you need consent to do that, like, in your initial intake with Kaiser or, you know, how every so often, is your contact information up to date or all that. And you, like, say can we contact you based on family member information, like, would it be okay if we reached out to you if we find something that might be pertinent to you based on a family member. (proband)
Direct contact programs are a complement to, not replacement of, patient-led familial sharing.	I don’t know about you guys, but I think the family is more powerful in urging their family members. It’s a shared condition we all have. We should think about our kids and our grandkids, so that argument is pretty strong. With the science backed up with Dr. Lee. 1 (proband)

**Table 3:** Design requirements for health system-led direct contact programs.

Theme	Exemplar Quote
Patients should provide consent for a provider to contact their relatives.	You have to obviously get permission from that patient and have that patient list every single family member and then yeah, that’s fine, but you couldn’t do it without permission. (relative)
Relatives should be able to decide which (if any) information they want to receive, and how they act on that information.	I may not want to know at all. I don’t want to carry the burden of knowing that there’s a possibility that they have the gene, right? (relative)
Multiple communication channels and multiple efforts should be used to communicate with relatives, with clear communication of the final outreach.	And here’s what—I really wouldn’t care. Somebody calls and tells me there’s a chance you may have a higher risk. Do with this what you want. That’s really what the information is at the end of the day…I don’t care if it was the medical assistant or the receptionist at the front... So I don’t think it really does matter as long as you’re getting the message out there to the most people. (relative)
Communication points with relatives: the patient has given their permission to contact; a clear reason for contact, information on the potential inherited risk and associated diseases. Information on cost and coverage for testing and information on relevant privacy and nondiscrimination laws may also be relevant.	The counselor just leaves a message and says this is regarding a condition that runs in your family related to cancer. I have spoken with your sister Molly. She gave me permission to share this information. You know, this is why it’s important for you. This is why it’s important for your daughters. If you want more information, please feel free to call me back or you can talk to your own doctor, et cetera. (proband)
The clinician should make a clear recommendation for genetic testing and provide clear follow-up steps.	This mutation runs in your family. Here are the risk factors. Here’s your percentage of, you know, getting this or whatever—end of story. And then you—here’s what we recommend. We recommend that you have testing, and then if you have children, your children have testing. This can occur in males and females. And then that’s it. (proband)
Patients should still receive support or written resources to share with their relatives	The packet from Group Health was fantastic. It had everything there. I didn’t have to run the risk of saying something wrong. I just sent the packet [to my relative]. Everything was there including my particular BRCA mutation and everything they needed to know. (proband)

## Data Availability

Requests for use of data presented in this study should be directed to the corresponding author.
